# Controlled in-cell activation of RNA therapeutics using bond-cleaving bio-orthogonal chemistry†
†Electronic supplementary information (ESI) available. See DOI: 10.1039/c7sc01380a



**DOI:** 10.1039/c7sc01380a

**Published:** 2017-06-14

**Authors:** Irfan Khan, Leah M. Seebald, Neil M. Robertson, Mehmet V. Yigit, Maksim Royzen

**Affiliations:** a Department of Chemistry , University at Albany , State University of New York , 1400 Washington Avenue , Albany , New York 12222 , USA . Email: myigit@albany.edu ; Email: mroyzen@albany.edu ; Tel: +1-518-442-3002 ; Tel: +1-518-437-4463; b The RNA Institute , University at Albany , State University of New York , 1400 Washington Avenue , Albany , New York 12222 , USA

## Abstract

Temporal control of siRNA activation is a major challenge for RNAi-based therapeutics. The majority of the reported siRNA delivery systems rely on environmental factors, such as differences in extracellular and intracellular redox potential, ATP concentration, or pH to activate an siRNA payload. However dynamic endogenous environments are far too complex to rely on for controllable siRNA release and can result in premature siRNA activation prior to reaching the intended biological target. In addition, there are uncertainties about timing, degree and rate of the siRNA activation with spontaneous release approaches. Herein we describe a bio-orthogonal chemistry approach to address this important challenge. With our approach we were able achieve two major goals: complete siRNA inactivation upon immobilization of the payload on the surface of iron oxide nanoparticles and controlled in-cell activation with the addition of a small non-toxic chemical trigger after sufficient cellular uptake of the nanoparticles was confirmed. We have demonstrated our in-cell activation approach using two siRNAs against green fluorescent protein (GFP) and cyclin dependent kinase 8 (CDK8) in GFP expressing MDA-MB-231 cell line. We anticipate that this methodology will potentially advance the clinical translation of RNAi-based therapeutics, as the described bio-orthogonal chemistry can be generalized for any siRNA of choice.

## Abstract 2

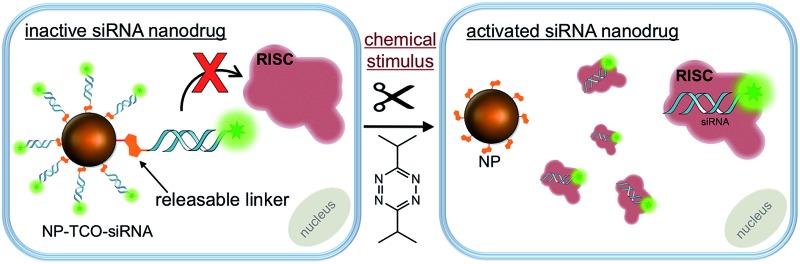
 *In vitro* exogenous activation of siRNA nanodrug using bio-orthogonal de-click chemistry.

## 

RNA interference (RNAi) is an evolutionarily conserved biological process for sequence-specific silencing of gene expression. Over a decade ago, synthetic small-interfering (si)RNAs have emerged as a promising therapeutic tool for post-transcriptional gene silencing in mammalian cells owing to their unique properties, such as stringent target-gene specificity, low immunogenicity, and simplicity of design.^1^ siRNA molecules are not cell permeable due to large size (∼14 kDa) and hydrophilicity (∼40 negative charges) and one of the main focuses of both academic and industrial research has been to achieve their delivery across plasma membranes.^2^ Numerous constructs have been reported towards achieving that goal, including strategies involving lipid encapsulation, cholesterol-conjugation, chemical modification, and attachment to different types of cell permeating nanoparticles.^3–9^ Though cell or tissue specific targeting has been the primary focus for the translation of RNAi into therapeutics, there are other challenges that need to be as seriously considered.^10–17^

One of the key elements of siRNA delivery that has been overlooked is temporal control over siRNA's activation.^18^ The majority of the RNAi-based therapeutic approaches rely on environmental factors, such as differences in extracellular and intracellular redox potential, ATP concentration, or pH to activate the conjugated or encapsulated siRNA payloads.^19–21^ However, these factors are highly heterogeneous in complex biological systems and can potentially result in premature activation of siRNA at unintended sites. An ideal delivery construct would allow precise temporal control over siRNA activation at a specific time point with an exogenous trigger, only after the delivery to the target cells or tissues has been confirmed.

Presently, photo-irradiation is the principal approach towards achieving temporal control over siRNA's activation.^22^ Photo-caging of siRNA has been described through chemical nucleobase modification with photo-labile moieties where the cells were transfected with chemically modified siRNAs and the internalized siRNAs were activated by release of photo-labile moieties upon photo-irradiation.^23,24^ Despite of a precise external control over *in vitro* gene-silencing events, the photo-caging approach has limitations towards clinical translation due to poor tissue penetration of light. Herein, we describe a bio-orthogonal chemistry-based approach to: (a) deactivate siRNA molecules by attaching them to a nanoparticle-based delivery vehicle, and (b) efficiently activate siRNA upon addition of a cell permeable small molecule chemical trigger. An important advantage of the bio-orthogonal chemistry strategy is that it relies on a cell permeable, small, and bio-inert molecule which can circulate in the body and release the siRNA payload from a nanoparticle vector regardless of the depth of the target tissue.

The siRNA delivery vehicle used in this study are biodegradable and biocompatible dextran coated superparamagnetic iron oxide nanoparticles (NP, ∼27 nm diameter) derived from clinically assessed MRI-active nanoparticles.^25^ The dextran polymer coating of the NP offers hundreds of bioconjugation sites through its amine termini and improves its water-solubility, dispersity, bioavailability and circulation half-life for *in vivo* studies.^26,27^ The key element of the NP construct is the bio-orthogonal chemistry-responsive linker which is utilized to covalently attach, and therefore inactivate, the siRNA payload on the NP surface, Scheme 1. In order for RNAi to occur, the siRNA molecules have to bind to and be incorporated into the multi-protein RNA Interference Specificity Complex (*RISC*).^28^ However, steric hindrance of the NP surface can prevent the siRNA, embedded in the dextran's polymer coating, from binding to the *RISC*.^4^ On the other hand, bio-orthogonal release of the siRNAs from the polymer surface can grant their accessibility to *RISC* thus initiating RNAi, Scheme 1.

**Scheme 1:**
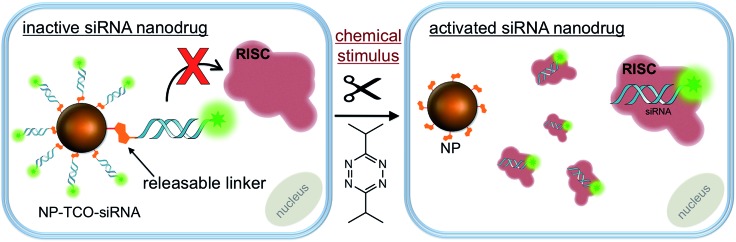
Schematic illustration of *in vitro* activation of **NP-TCO-siRNA** (prodrug) with an external chemical trigger. The siRNA conjugated on NP surface cannot access to *RISC* and silence the target genes. Addition of tetrazine results in siRNA release from the nanoparticle surface. The activated siRNA can access to the *RISC* to initiate RNAi.

## Results and discussion

**Fig. 1:**
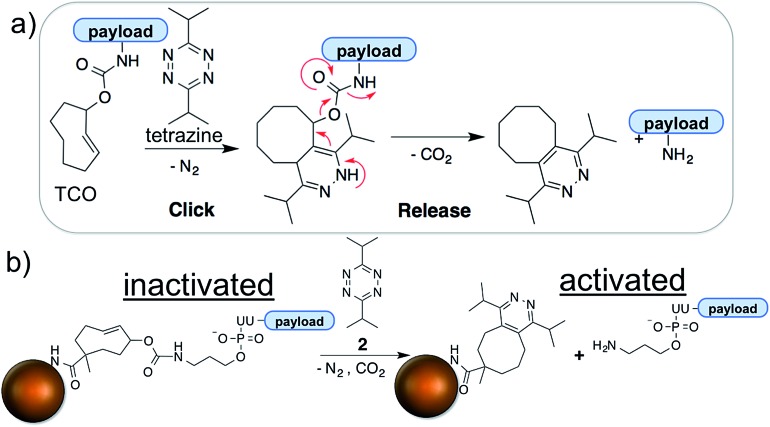
Mechanism of IEDDA chemistry and tetrazine triggered siRNA activation using bio-orthogonal chemistry. (a) The underlying mechanism of the IEDDA chemistry involves a click step, which is followed by the spontaneous tautomerization and release of the payload attached at the allylic position of TCO; (b) siRNA is immobilized to the nanoparticle surface through the heterobifunctional TCO. The TCO-facilitated immobilization completely shuts down the activity of siRNA which is activated only upon addition of tetrazine, a chemical stimulus highly specific to TCO.

The bio-orthogonal activation of siRNA payloads is based on an inverse electron demand Diels–Alder (IEDDA) reaction between *trans*-cyclooctene (TCO) and tetrazine.^29,30^ The two reactive groups are highly specific for and reactive with each other. The two-step reaction is termed bio-orthogonal because it readily proceeds in a physiological environment (aqueous solution, neutral pH, *etc.*) on a minute timescale and is unreactive to functional groups present in the cell, such as amines, alcohols, thiols, phosphates, *etc.* ^29,30^ Thus, the chemistry is inert to the endogenous biochemical environments and can be initiated only by an exogenous chemical trigger; tetrazine. The products of the reaction are non-toxic and exhibit minimal interference with cell metabolism. The IEDDA chemistry has found numerous applications ranging from drug delivery, to site-specific labeling of biomolecules, to radio-imaging of cancerous tissues.^31–33^ We and others have further modified the IEDDA chemistry to adapt it for bio-orthogonal ‘de-click’ reactions using functionalized allylic carbamates (Fig. 1a), which can be triggered to undergo self-immolative decomposition following the cycloaddition reaction and subsequently release the attached payload (illustrated in Fig. 1a), as well as CO_2_.^27,34,35^

**Scheme 2:**
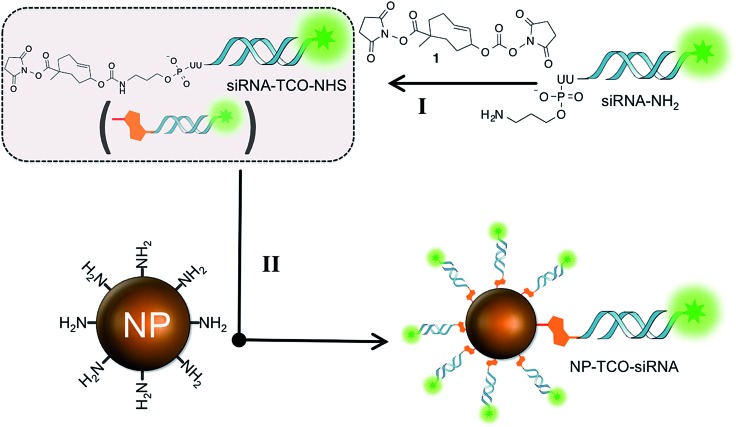
Stepwise synthesis of **NP-TCO-siRNA** (prodrug) from **siRNA-NH_2_** and NP-NH_2_. The siRNA is functionalized with heterobifunctional TCO **1** at the allylic position *via* a releasable carbamate linker and NP *via* a non-releasable amide bond.

Towards this goal, we synthesized a heterobifunctional TCO **1**, shown in Scheme 2 and Fig. S1,† and modified it with siRNA at the allylic position *via* a releasable carbamate linker and NP *via* a non-releasable amide bond (Fig. 1b). The RNAi experiments described below illustrate that while immobilized to the NPs, the siRNAs remain inactive due to steric hindrance from the nanoparticle surface. Addition of the bio-orthogonal partner **2**, results in a cycloaddition product that can further undergo tautomerization leading to separation and release of the siRNA from NPs (Fig. 1b). The released siRNA becomes fully activated towards silencing specific genes. This chemistry is the foundation of controlled siRNA activation approach demonstrated for two model siRNA systems described below.

A well characterized and easy-to-study RNAi system against the green fluorescent protein (GFP) gene^6^ was utilized to demonstrate efficacy of (a) siRNA inactivation due to immobilization on NP surface, followed by (b) activation *via* the bio-orthogonal IEDDA reaction with an exogenous tetrazine trigger, Fig. 1b. The siRNA against GFP gene was immobilized on NP surface using the heterobifunctional TCO linker **1** coupled to either guide or passenger strand of the siRNA molecule (Fig. S2 and S3†). Both **NP-TCO-siRNA** constructs were assembled by stepwise coupling chemistry, schematically illustrated in Scheme 2. The primary amine group of the siRNAs was chemically modified with the heterobifunctional TCO **1**. The resulting double stranded RNAs were coupled to the amine termini of NPs, resulting in **NP-TCO-siRNA** constructs against GFP. The conjugation of siRNA to NP was validated by an increase in the hydrodynamic radii of the NPs, fluorescence and absorbance spectra of NPs before and after conjugation with cy5.5-labeled siRNA, Fig. S9.† The average number of siRNA per NP was determined to be about 12 by calculating the cy5.5 per NP with absorbance spectra; consistent with previous studies using a similar type of nanoparticles.^26^ Complete release of siRNA-cy5.5 cargo upon addition of tetrazine was also validated by UV-Vis spectra, Fig. S10.†

**Fig. 2:**
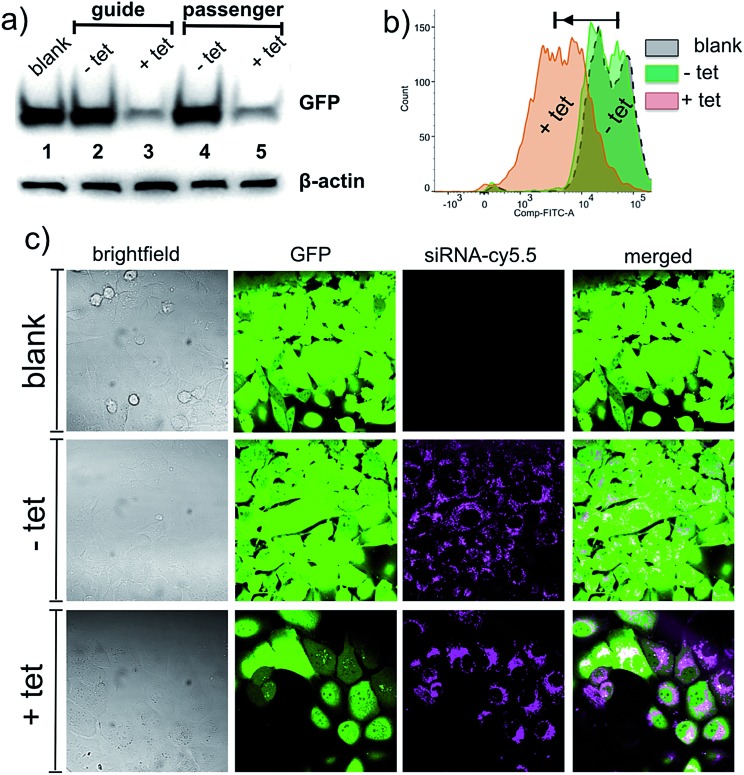
Western blot analysis, flow cytometry and fluorescence microscopy measurements showing knockdown of GFP in MDA-MB-231-GFP cells. (a) Western blotting analysis of GFP levels, β-actin was used as a loading control; immobilization of siRNA on NP surface either through guide or passenger strand shuts down the activity of siRNA (lanes 2 and 4 *vs.* 1) whereas the addition of the tetrazine **2** activates the siRNA resulting in a remarkable decrease in the expression of GFP (lanes 3 and 5). (b) Flow cytometry analysis shows a decrease in the fluorescence of GFP signal upon addition of the tetrazine **2** (c) confocal fluorescence microscopy demonstrates that the siRNA (cy5.5) is taken up by cells and the loss in the GFP signal is observed with the addition of the tetrazine **2**. The siRNAs tend to diffuse in the cell upon addition of the tetrazine **2**, suggestive of release from the **NP-TCO-siRNA**.

The initial silencing experiments were carried out using GFP expressing MDA-MB-231 cell line. The cells were treated with **NP-TCO-siRNA** for 48 h and the GFP expression was analyzed using western blot, confocal fluorescence microscopy, and flow cytometry. For the western blot experiments, β-actin was used as a reference to evaluate the knockdown of GFP. The western blot analysis (Fig. 2a), measuring GFP protein level in the extracted cells, demonstrated that the function of siRNA was completely inactivated when immobilized on the NP surface either through guide or passenger strand. In fact, GFP expression was observed to be on the same level as in the untreated cells (blank). Though this was the goal of the study, such a significant degree (∼100%) of inactivation was intriguing. To demonstrate that siRNA can be released from NPs and thereby activated in the cellular environment, the cells were incubated with 10 μM of tetrazine **2**, 48 h after the **NP-TCO-siRNA** treatment. The western blot analysis indicated 95% decrease in GFP expression level upon addition of tetrazine. Both siRNA immobilization strategies, through either 3′-uridine of passenger or guide strands, displayed analogous outcomes indicating that similar degree of inactivation/activation can be achieved regardless of the choice of strand for attachment. In order to demonstrate that the activation of siRNA is highly specific to tetrazine addition, the cells were treated with a tetrazine precursor; 4-isopropylbenzonitrile; which didn't decrease the GFP fluorescence, Fig. S6.†

Flow cytometry analysis, described in Fig. 2b, is supportive of the western blot analysis and the microscopy data described below. The population of **NP-TCO-siRNA** treated cells (**–tet**, green) with a strong GFP signal is practically identical to the population of the GFP-expressing untreated cells (gray). A significant fraction of the cell population (∼55%) lost its inherent GFP fluorescence upon addition of the tetrazine **2** (**+tet**). Based on the western blot analysis, the loss of fluorescence is attributed to the result of silencing of the GFP gene by the activated siRNA.

Confocal fluorescence microscopy studies were performed to monitor **NP-TCO-siRNA** uptake and silencing of GFP. Fig. 2c shows MDA-MB-231 cells treated with **NP-TCO-siRNA** for 48 h, followed by another 48 h incubation; for RNA interference to take place;^6^ in fresh media lacking (**–tet**) or containing (**+tet**) the tetrazine **2**. In agreement with the western blot and flow cytometry data, Fig. 2a and b, cells treated with **NP-TCO-siRNA** alone displayed similar levels of GFP fluorescence, as the untreated cells. Addition of the bio-orthogonal trigger, tetrazine, resulted in a remarkable loss of the GFP signal as hypothesized. A series of control experiments using confocal microscopy showed that siRNA and NP mixture without any covalent attachment, could not affect GFP fluorescence due to the fact that the cells cannot be transfected with siRNA without a vector attached to it (Fig. S5†). Control experiments also showed that tetrazine (10 μM) itself has no effect on GFP's fluorescence (Fig. S5†), thus confirming that **2** acts *via* IEDDA chemistry releasing and activating the siRNA inside the cells.

Fluorescence microscopy was also utilized to study **NP-TCO-siRNA** internalization and its fate upon activation *via* tetrazine. Punctate staining observed in the cy5.5 emission channel (Fig. 2c, **–tet**) indicates that **NP-TCO-siRNA** was efficiently taken up and localized inside the cells. Addition of the tetrazine **2** resulted in a more diffused staining, shown in (Fig. 2c, **+tet**), which we attribute to the release and departure of the activated siRNA from localized nanoparticle environments within the cell.

To illustrate the generality of the triggered siRNA release paradigm, we have adapted our approach towards silencing the CDK8 gene, which has been shown to play important biological roles in a number of cancers.^36–39^ CDK8 is an oncoprotein that promotes development and progression of cancer.^38^ Knockdown of CDK8 in MDA-MB-231 breast cancer cells has been shown to reduce the cell proliferation.^40^ Therefore, triggered knockdown of CDK8 in MDA-MB-231 breast cancer cells is an ideal model to demonstrate the therapeutic potential of our bio-orthogonal chemistry approach for controlled RNAi therapeutics.

**Fig. 3:**
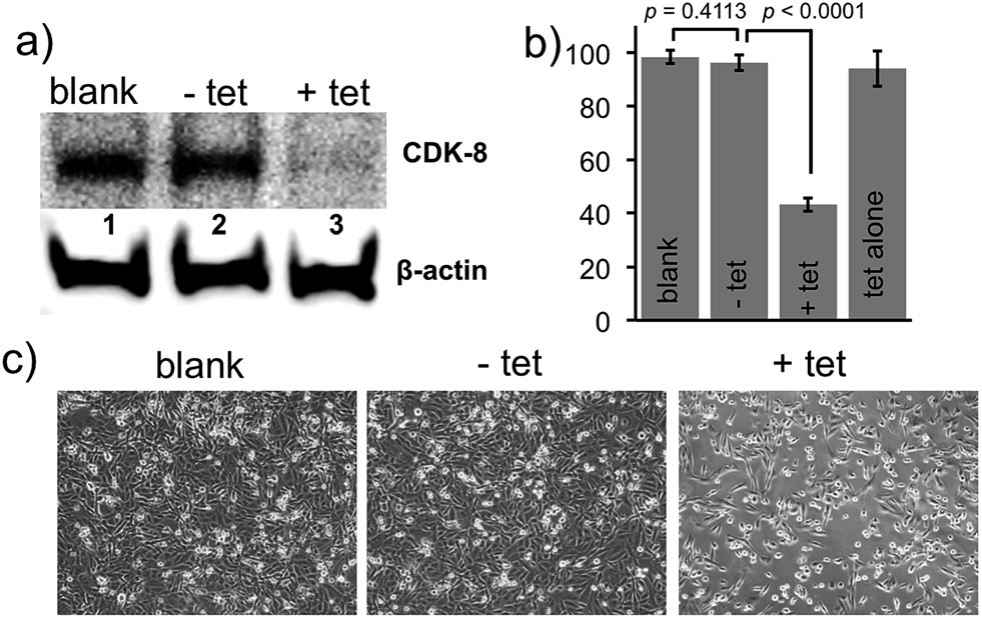
Western blotting analysis of CDK8 protein, MTT analysis and bright field microscopy measurements in MDA-MB-231 cells. (a) Western blotting analysis of CDK8 levels. Immobilization of siRNA on NP surface shuts down the activity of siRNA (lanes 2 *vs.* 1) whereas addition of the tetrazine **2** activates the siRNA resulting in a remarkable decrease in the expression of CDK8 (lanes 3 *vs.* 1). β-Actin was used as a loading control. (b) Relative levels of cell proliferation measured by MTT assay; activated siRNA slows down proliferation of MDA-MB-231 cells due to knockdown of the CDK8 gene. (c) Brightfield image illustrating decreased population of MDA-MB-231 cells after silencing of CDK8 gene with the activation of prodrug using tetrazine.

We tested siRNA against the CDK8 gene with a primary amine modification on the 3′-uridine of the passenger strand (**siRNA-CDK8**, materials and methods). Following the synthetic approach described above, the amine group was chemically modified with the heterobifunctional TCO **1**, Fig. S4.† The resulting double stranded RNA was coupled to the amine termini of NPs, resulting in a **NP-TCO-siRNA** prodrug against CDK8. The MDA-MB-231 cells were treated with the prodrug for 48 h, followed by fresh media either containing (**+tet**) or lacking (**–tet**) tetrazine. After additional 48 h, the cells were lysed and CDK8-expression was analyzed by western blot. As illustrated in Fig. 3a, in agreement with our hypothesis, the prodrug was unable to affect the expression of the protein. On the other hand, addition of the tetrazine **2** resulted in a complete knockdown of CDK8 (Fig. 3a) according to western blot analysis.

MTT assay was performed to evaluate relative cell growth. Fig. 3b draws the comparison between proliferation of the untreated cells, as well as the cells treated with either prodrug alone (**–tet**) or prodrug and **2** (**+tet**). As predicted, addition of the bio-orthogonal trigger resulted in marked decrease of cellular growth due silencing of CDK8. In a control experiment, we have established that addition of the tetrazine alone had no effect on cell proliferation. Bright-field cell images, shown in Fig. 3c, also confirmed slower proliferation of the MDA-MB-231 cells upon triggered siRNA activation. These results demonstrate the generality and also the therapeutic potential of siRNA inactivation due to immobilization on NP surface and activation *via* the bio-orthogonal IEDDA reaction with an exogenous trigger. All of the *in vitro* studies were carried out using 10 μM of tetrazine which did not display any notable cytotoxicity confirmed by cell viability studies and bright-field images, Fig. S7 and S8.†

## Conclusion

In conclusion we have developed a robust and versatile system that allows temporal control of RNAi activation using an exogenous bio-inert small molecule trigger. The nanoparticle template is derived from clinically assessed *T*_2_-weighted MRI-active nanoparticles,^25^ which were also explored as delivery vehicles for therapeutic macromolecules.^6^ The key element of the design is a bio-orthogonal linker that is utilized for immobilization of siRNA on the nanoparticle surface. We have shown that siRNAs can be immobilized through either guide or passenger strands and their activity can be shut down completely. Steric environment of the polydextran coating results in a complete deactivation of siRNA, which is unable to access *RISC* and silence the target gene. Meanwhile, fluorescent label allows monitoring cellular uptake. Once the uptake has been confirmed, addition of a bio-orthogonal trigger results in a bond-cleaving cascade that releases siRNA from the nanoparticle surface. The activated siRNAs against GFP and CDK8 gene have shown exceptional activity towards their intended targets. The latter was able to silence the intended oncogene and slow proliferation of breast cancer cells. We envision that the described construct hold great potential for clinical translation, as it can be explored towards controlled activation of any siRNA of interest.

## Materials and methods

### Materials

All chemicals were received from commercial sources and used without further purification. Chromatographic purifications of synthetic materials were conducted using SiliaSphere™ spherical silica gel with an average particle and pore size of 5 μm and 60 Å, respectively (Silicycle Inc, QC, Canada). Thin layer chromatography (TLC) was performed on SiliaPlate™ silica gel TLC plates with 250 μm thickness (Silicycle Inc, QC, Canada). Preparative TLC was performed using SiliaPlate™ silica gel TLC plates with 1000 μm thickness. HPLC purification was performed using Phenomenex Luna 5u C18 (2) semi-preparative column (250 × 10 mm). ^1^H and ^13^C NMR spectroscopy was performed on a Bruker NMR at 400 MHz (^1^H) and 100 MHz (^13^C). All ^13^C NMR spectra were proton decoupled. Fluorescence microscopy experiments were carried out using Zeiss LSM 710 Pascal laser confocal microscope (Carl Zeiss Microscopy, Thornwood, NY, USA). Image acquisition and analyses were performed using Zeiss ZEN 2012 Confocal Microscopy Software (Release 2.02). Flow cytometry experiments were carried out using FACS Aria III instrument (BD Biosciences, San Jose, CA, USA) and analyzed using FlowJo software (Ashland, OR, USA). Nanoparticle purifications were done using Sephadex PD-10 columns, purchased from GE Healthcare (Chicago, IL, USA). For ESI-MS analysis, the siRNA was desalted through 3–5 cycles of buffer exchange using mass spectrometry grade ammonium acetate (Sigma-Aldrich, St. Louis MO) and 3 KDa centricon (EMD Millipore, Billerica MA, USA). A rabbit polyclonal antibody specific for CDK8, rabbit monoclonal antibody to GFP, rabbit monoclonal antibody for β-actin and horseradish peroxidase-conjugated secondary antibodies, used for chemiluminescence, were obtained from Cell Signaling Technology, (Danvers, MA, USA). All siRNAs were purchased from Dharmacon, GE (Lafayette, CO, USA) with following sequences and modifications;

siRNA-GFP1:

5′-GCAAGCUGACCCUGAAGUUCUU-**N3**-3′ (passenger)

3′-UUCGUUCGACUGGGACYUCAAG-5′ (guide)

siRNA-GFP2:

5′-GCAAGCUGACCCUGAAGUUCUU-**cy5.5**-3′ (passenger)

3′-**N3**-UUCGUUCGACUGGGACYUCAAG-5′ (guide)

siRNA-CDK8:

5′-GGGAAUGGUGAAGUCACUAUUAUAUUU-**N3**-3′ (passenger)

3′-UUCCCUUACCACUUCAGUGAUAAUAUA-5′ (guide)

### Methods

#### Cell culture

MDA-MB 231 cells were purchased from ATCC (American Type Culture Collection, cat.# HTB-26) and MDA-MB-231/GFP cells were purchased from Cell Biolabs, Inc. (San Diego, CA, USA, Ref.# AKR-201) and propagated in Dulbecco's modified Eagle's medium (DMEM) containing 5% fetal bovine serum (FBS), supplemented with 100 U mL^–1^ penicillin, and 100 μg mL^–1^ streptomycin at 37 °C in a 5% CO_2_ incubator.

#### Synthesis of dextran-coated magnetic nanoparticles (NPs)

The dextran coated iron oxide nanoparticle synthesis protocol was adapted from the protocol that others and we published previously.^25,26,41–43^ Briefly, (18 g) of Dextan-T10 (Pharmacosmos, Holbaek, Denmark) was mixed in 60 mL of double-distilled water and stirred in a round bottom flask at 4 °C. FeCl_3_·6H_2_O (1.3 g) was added while flushing nitrogen gas into the reaction mixture. FeCl_2_·4H_2_O (0.8 g) was added into the mixture and then 30 mL of concentrated cold NH_4_OH (∼28%) was added to the stirring mixture. The temperature was increased to 75–85 °C for an hour to induce formation of nanoparticles. The mixture was cooled to room temperature and concentrated to 40 mL using Amicon Ultra centrifugal units (MWCO 100 kDa; Millipore, Billerica, MA, USA). The dextran coating on the nanoparticles was cross-linked with the addition of 70 mL of 5 M NaOH and 28 mL of concentrated epichlorohydrin and the mixture was stirred for 8 hours. 120 mL of concentrated NH_4_OH was added to the stirring mixture. The nanoparticle solution was purified using a dialysis bag (MWCO 14 kDa) against water and suspended in 40 mM citrate buffer (pH 8.0). The nanoparticle concentration was determined based on iron concentration (10.8 mg mL^–1^ Fe) and measured UV spectroscopy. The size of the nanoparticles (∼25 nm diameter) was determined by using dynamic light scattering (DLS) DynaPro Titan (Wyatt technology Corporation, USA). The number of amine groups was determined according to the procedure described previously.^42,43^

#### Modification of siRNA with the heterobifunctional TCO

The aforementioned double stranded siRNAs were dissolved in nuclease-free H_2_O to prepare 2 mM stock solutions. The heretobifunctional TCO **1** was synthesized based on procedures published in the literature and characterized by ^1^H NMR and ESI-MS.^34^ A 1.5 M stock solution of the heterobifunctional TCO **1** was prepared in DMSO. The 150 μL of siRNA suspension was added to 540 μL of 50 mM sodium borate buffer (pH 9.5). Immediately after, 10 μL of TCO **1** was added and the resulting solution was agitated for 10 h at room temperature. The TCO-modified siRNA was concentrated using centricon filters (3 KDa, EMD Millipore, Billerica MA, USA) and purified by ethanol precipitation and characterized by ESI-MS analysis, Fig. S2–S5.† Yields ranged from 12% to 15%.

#### Synthesis of **NP-TCO-siRNA**s

The concentration of TCO-modified siRNAs in sodium borate buffer was adjusted to 20 mM using centricon filters. 50 μL of the TCO modified siRNA stock solution was mixed with 0.5 mL of NPs (10.8 mg mL^–1^ Fe) in 20 mM citrate buffer (pH 8.0) and agitated for 24 h. The buffer of the **NP-TCO-siRNA** was exchanged by cell culture grade PBS buffer (pH 7.4) using Sephadex PD-10 columns.

#### Treatment of MDA-MB-231/GFP breast cancer cells with **NP-TCO-siRNA**

The cells were treated with final concentration of 50 nM of **NP-TCO-siRNA** conjugate for 48 h (step 1). Cellular nanoparticle internalization was monitored and recorded by confocal fluorescence microscopy. The cells were subsequently treated with DMEM either containing or lacking 10 μM tetrazine **2** (step 2) for additional 48 h. Knock-down of GFP was monitored and confirmed by fluorescence microscopy, flow cytometry, and western blotting.

#### Confocal fluorescence microscopy

Confocal fluorescence microscopy was performed using GFP expressing MDA-MB-231 breast cancer cells. The cells (1 × 10^3^) were incubated with **NP-TCO-siRNA** for 48 h at 37 °C in poly-d-lysine coated glass bottom dishes (MatTek Corporation, Ashland, MA, USA). The cells were then washed three times with PBS, (pH 7.4). Fluorescence microscopy (GFP and cy5.5 channels) was performed to confirm nanoparticle uptake and GFP fluorescence. Subsequently, the cells were further incubated with and without the tetrazine **2** for additional 48 h. Image acquisition and analyses were performed using Zeiss ZEN 2012 Confocal Microscopy Software (Release 2.02). The final images were color-coded green for GFP and magenta for cy5.5.

#### Flow cytometric analysis

GFP expressing MDA-MB-231 cells were treated with **NP-TCO-siRNA** (48 h) and **2**, same as above. The cells were detached using trypsin, washed twice with 2% FBS/PBS and resuspended in 500 μL of 2% FBS/PBS before analysis. Data from 10^6^ cells were acquired using a FACS Aria III cell sorter equipped with a 488 nm/blue coherent sapphire solid-state laser, 20 mW (BD Biosciences, San Jose, CA, USA). Data analyses were carried out using FlowJo software (Ashland, OR, USA), according to manufacturer's instructions. Parameters, such as MFI and the percentages of specific populations, were quantified by histogram analysis.

#### Immunoblotting of GFP and CDK8 proteins

Cells treated with 50 nM of **NP-TCO-siRNA** alone (**–tet**), or **NP-TCO-siRNA** and **2** (**+tet**) were lysed in cell lysis buffer; 150 mM NaCl, 50 mM Tris pH 8.0, 1 mM EDTA, 1% NP-40, 0.1% SDS, 1 mM phenylmethylsulfonyl fluoride, and complete protease inhibitor cocktail (Thermo Scientific, Rockford, IL). Typically, 4 μg of proteins was resuspended in 4X sample buffer (240 mM Tris pH 6.8, 4% SDS, 40% glycerol, 4% β-mercaptoethanol, 0.01% bromophenol blue) and boiled for 10 min. The proteins were resolved on 10 to 12% SDS-PAGE, followed by transfer onto a nitrocellulose membrane (Thermo Scientific, Rockford, IL). Following incubation with the respective primary antibody and horseradish peroxidase (HRP)-conjugated secondary antibody, proteins were visualized by an enhanced chemiluminescence detection method (Pierce™ ECL Western Blotting Substrate, Thermo Scientific, Rockford, IL).

#### Cell proliferation assay

The colorimetric MTT (3-(4,5-dimethylthiazol-2-yl)-2,5-diphenyltetrazolium bromide) assay protocol, adopted from previously published work, was used to evaluate proliferation of MDA-MB-231 cells.^41^ On day one, 1 × 10^3^ cells per well in 96-well plates in 100 μL DMEM were plated and incubated for 24 h. On day two, the medium was removed and the cells were treated with 50 nM of **NP-TCO-siRNA** against CDK8. On day four, the medium was replaced with 100 μL of fresh medium containing 10 μM tetrazine **2** and cells were incubated for additional 48 h. On day six, the medium was removed and cells were incubated with 100 μL of MTT solution (0.6 mg mL^–1^ in DMEM) per well. Cells were incubated in the dark for 4 h at 37 °C. At the end of the incubation, the MTT solution was then replaced with 100 μL of DMSO containing 4% aqueous ammonia per well and agitated for 30 minutes. The absorbance of the purple formazan was recorded using a BioTek Synergy HT multi detection microplate reader at 550 nm. Results were generated from at least three independent arrays of triplicate experiments.

#### Statistical analysis

Data were expressed as mean ± SD. Statistical differences were analyzed by the Student's *t*-test (; http://www.graphpad.com). A value of *P* < 0.05 was taken as statistically significant. MTT and caspase-3 experiments were performed in triplicate.

## Author contributions

MVY and MR designed the experiments. LS carried out the chemical syntheses, while IK performed the biochemical and the cell-based assays. NR helped with nanoparticle characterization. MVY and MR wrote the manuscript with input from all authors.
