# Supplementary material for: Controlled in-cell activation of RNA therapeutics using bond-cleaving bio-orthogonal chemistry
Source: Chem Sci. 2017 Jun 14;8(8):5705–12. doi: 10.1039/c7sc01380a (PMC5621156; doi:10.1039/c7sc01380a)
Supplement: Supplementary file 1 [file SC-008-C7SC01380A-s001.pdf]

Electronic Supplementary Material (ESI) for Chemical Science.
This journal is © The Royal Society of Chemistry 2017

Supplementary Information

Controlled in-cell activation of RNA therapeutics using bond-cleaving bio-orthogonal chemistry

Irfan Khan[†, ‡], Leah M. Seebald [†, ‡], Neil M. Robertson[†, ‡], Mehmet V. Yigit[†, ‡, *] and Maksim Royzen[†, ‡, *]

† Department of Chemistry,
University at Albany, State University of New York,
1400 Washington Avenue, Albany, New York 12222, United States.

‡ The RNA Institute,
University at Albany, State University of New York,
1400 Washington Avenue, Albany, New York 12222, United States.

*Correspondence:
Tel: (1) 518-442-3002
myigit@albany.edu

Tel: (1) 518-437-4463
mroyzen@albany.edu

Abstract

Here, we have listed the results of additional experiments and analysis of synthesized products as supplementary information for the manuscript.

Table of Content

1. Supporting Figures ………………………S3-S12

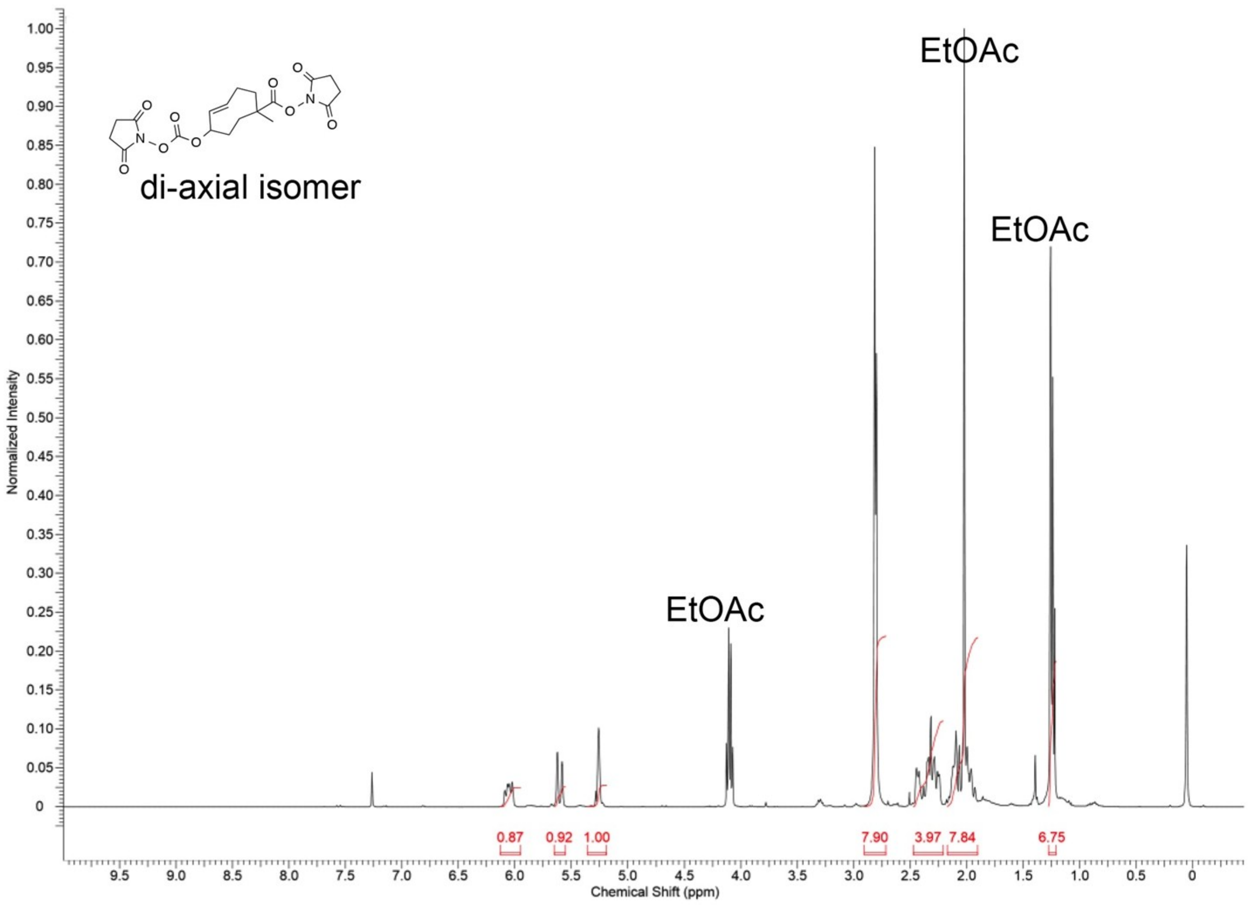

Figure S1. ^{1}H NMR spectrum of the heretobifunctional TCO **1** that was used to attach siRNA to NPs.

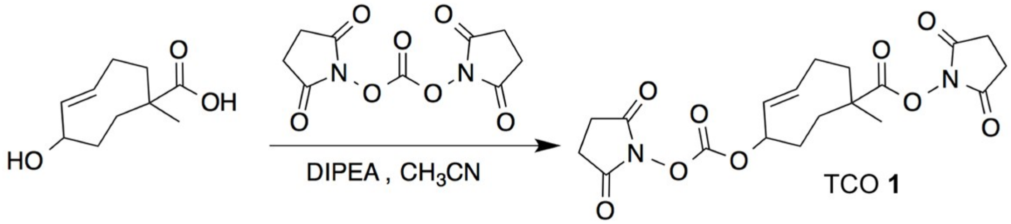

The heretobifunctional TCO **1** was synthesized based on procedures described in *Bioconjugate Chem.* ***2016****, 27, 1697-1706.* Yield: 81%. The obtained NMR spectrum matched the one reported.

^{1}H NMR ($CDCl_3$): δ 6.09-6.02 (m, 1H), 5.60 (d, J = 16.4 Hz, 1H), 5.26 (s, 1H), 2.81 (s, 8H), 2.44-2.24 (m, 4H), 2.12-1.93 (m, 8H)

HRMS (ESI) calcd. for $C_{19}H_{23}N_2O_9$ $[M+H]^+$ 423.1404; found 423.1387

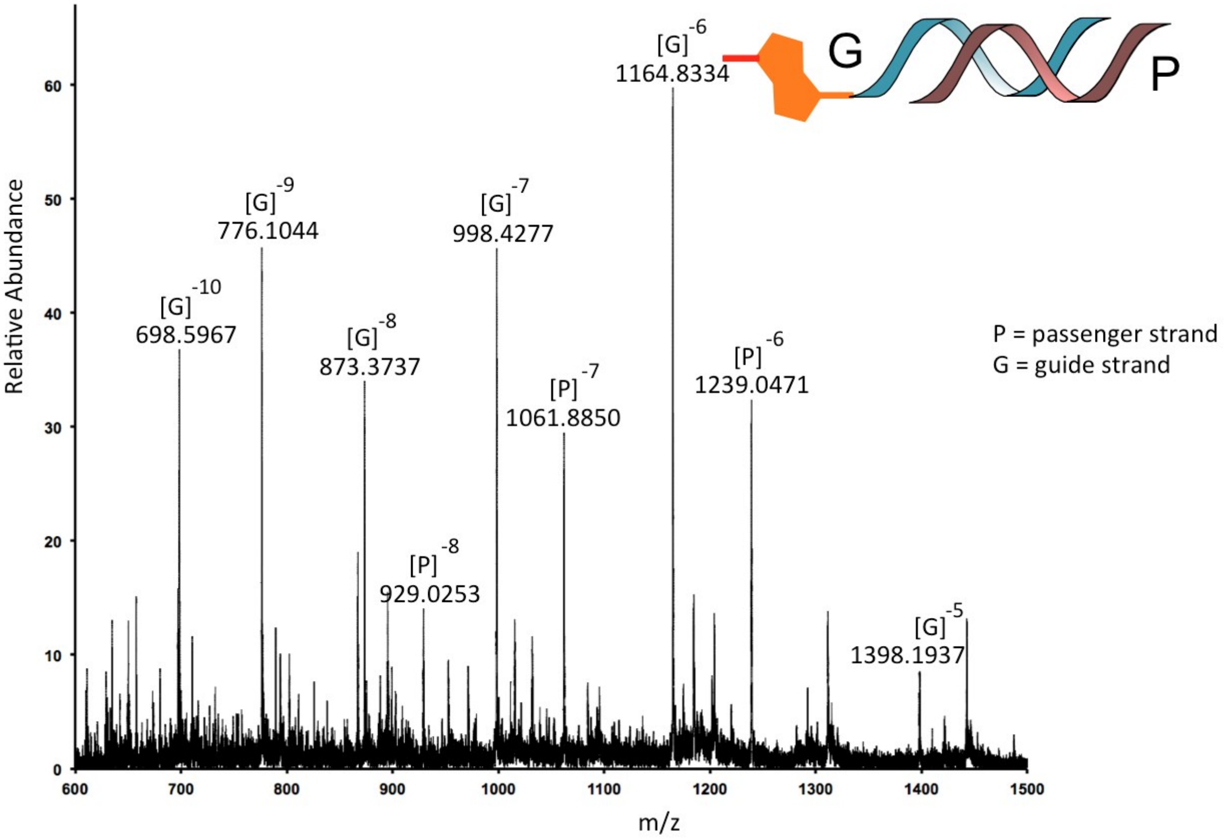

Figure S2. ESI-MS analysis of **siRNA-GFP1** modified with TCO.

List of the predicted peaks:

Charge State (n)	Guide Strand $[M]^{-n}$	Passenger Strand $[M+Na]^{-n}$
-5	1397.375	
-6	1164.311	1239.145
-7	997.837	1061.980
-8	872.981	929.107
-9	775.871	
-10	698.183	

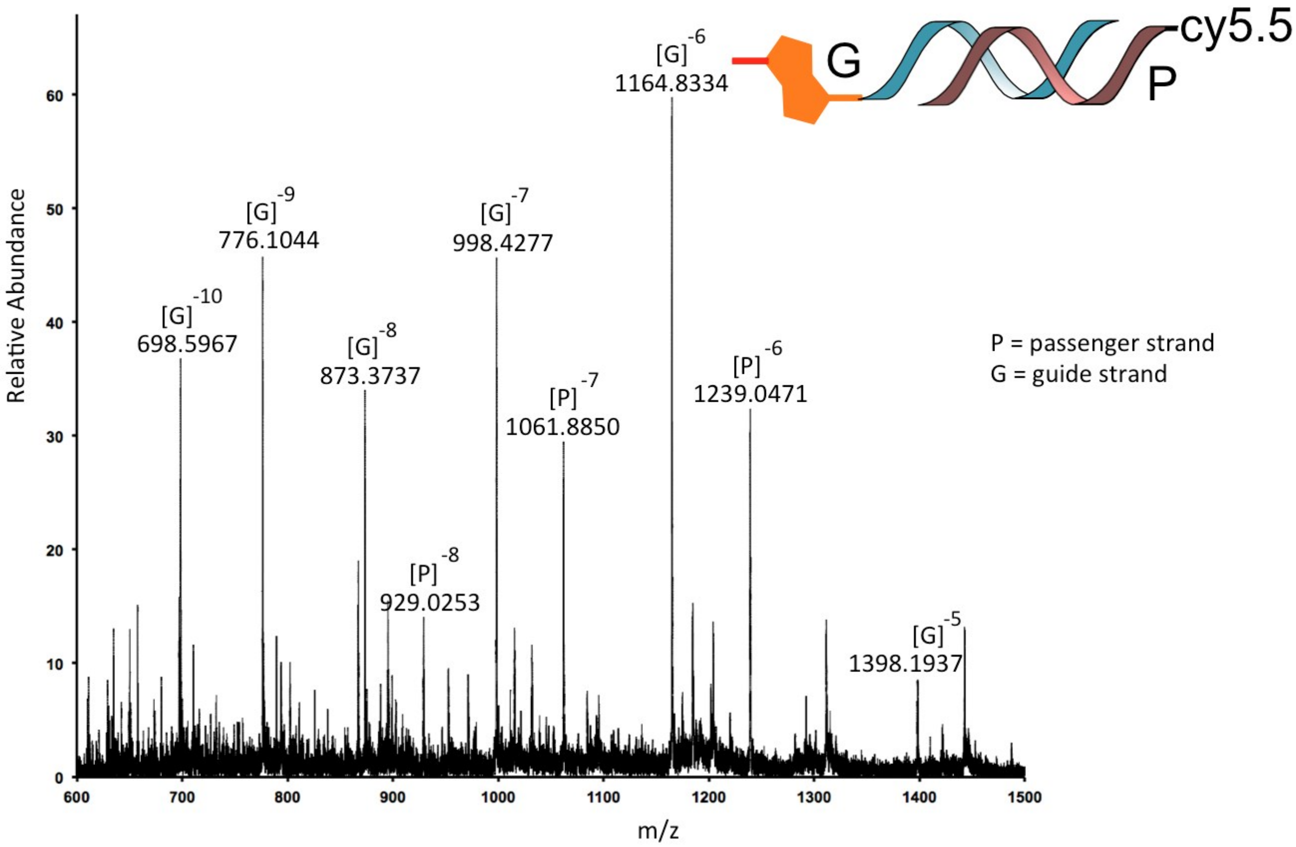

Figure S3. ESI-MS analysis of **siRNA-GFP2** modified with TCO.

List of the predicted peaks:

Charge State (n)	Guide Strand $[M+Na]^{-n}$	Passenger Strand $[M]^{-n}$
-4		1974.821
-5	1490.597	1579.655
-6	1241.996	1316.211
-7	1064.424	1128.037
-8	931.245	986.906
-9	827.662	877.138

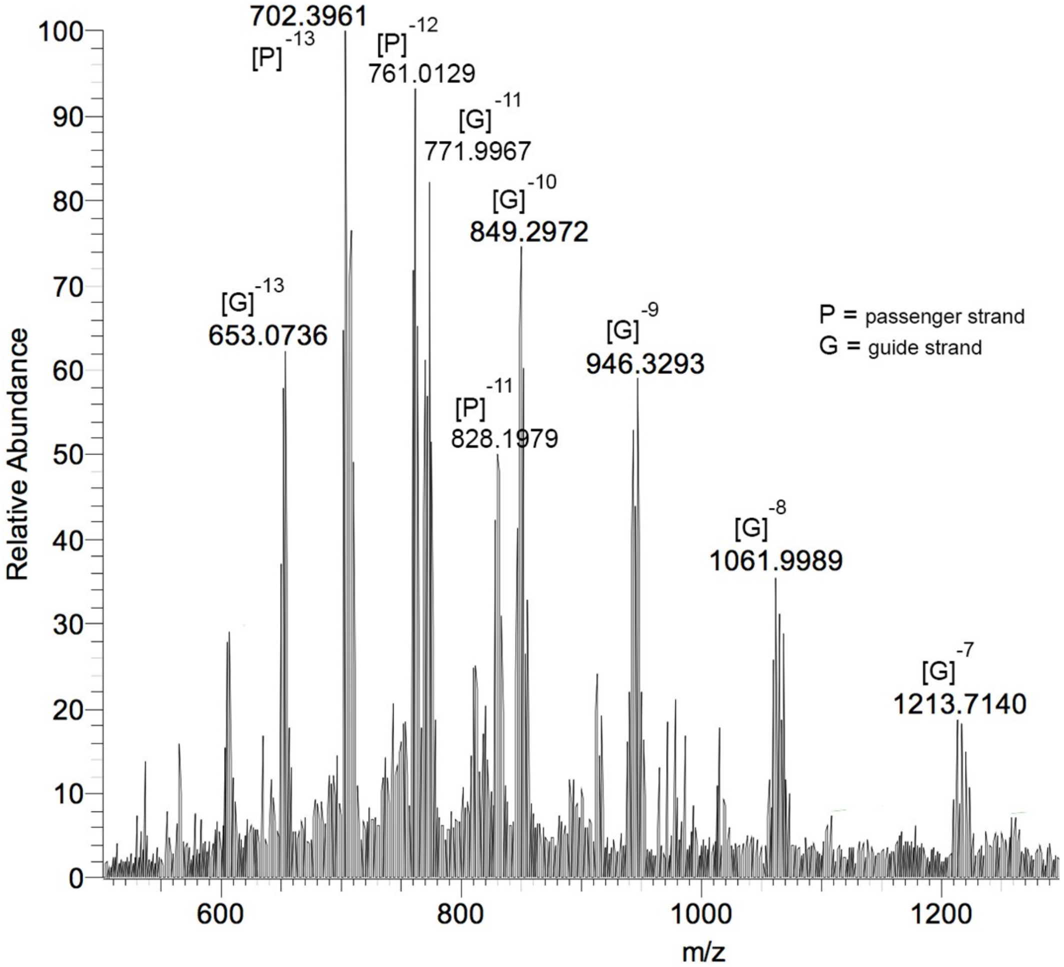

Figure S4. ESI-MS analysis of **siRNA-CDK8** modified with TCO.

List of the predicted peaks:

Charge State (n)	Guide Strand $[M+2Na]^{-n}$	Passenger Strand $[M+Na]^{-n}$	Passenger Strand $[M+2Na]^{-n}$
-7	1213.286		
-8	1061.499		
-9	943.443		
-10	848.997		
-11	771.724	828.286	
-12	707.330		761.010
-13	652.842		702.393

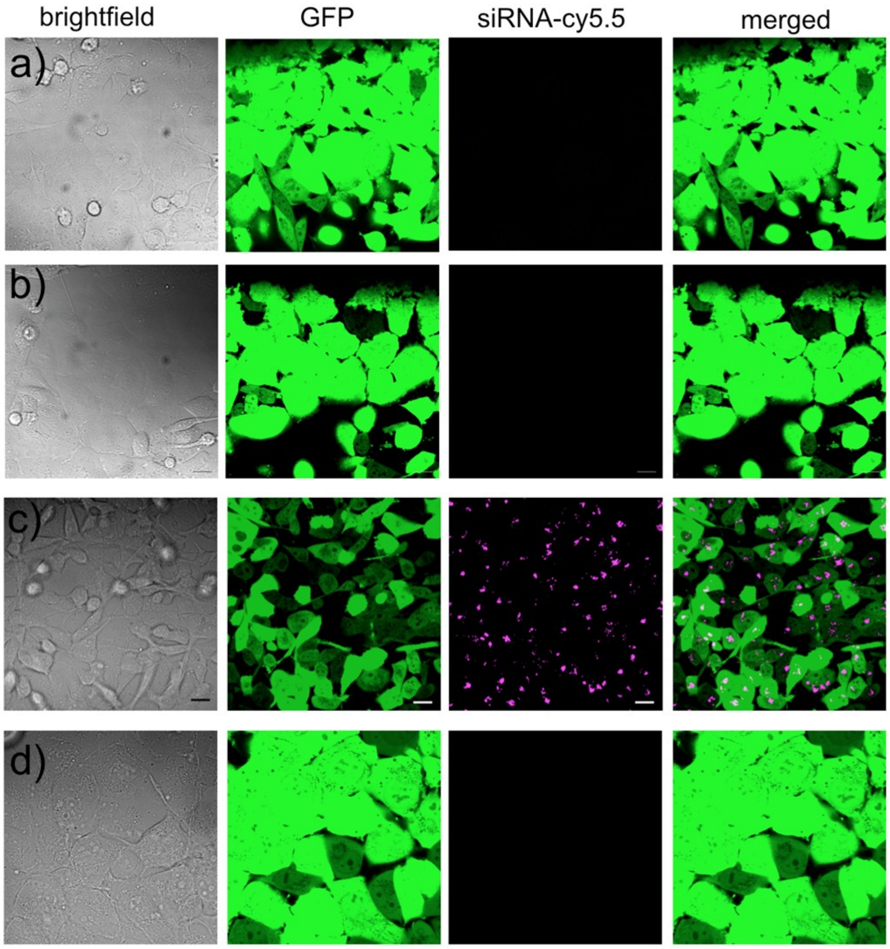

Figure S5. Control experiments for silencing GFP using **NP-TCO-siRNA**. a) *blank*: Untreated GFP expressing MDA-MB-231 cells showing a strong fluorescence in the GFP channel. b) *negative control:* GFP expressing MDA-MB-231 cells treated with a mixture of NPs and siRNA without a covalent attachment. The nonconjugated siRNA does not affect the GFP signal because it cannot pass through cellular membrane without a vector attached to it. c) *positive control:* Lipofectamine transfection of GFP expressing MDA-MB-231 cells with the **siRNA-GFP2** results in reduction of GFP signal which also suggests that the two chemical modifications (amine on guide and cy5.5 on passenger strands) do not interfere with RNAi. d) GFP expressing MDA-MB-231 cells treated with the tetrazine **2**. The latter does not affect the GFP signal suggesting that the loss in GFP signal with **NP-TCO-siRNA** and tetrazine is due to the release of siRNA upon bio-orthogonal reaction but not due to the tetrazine itself.

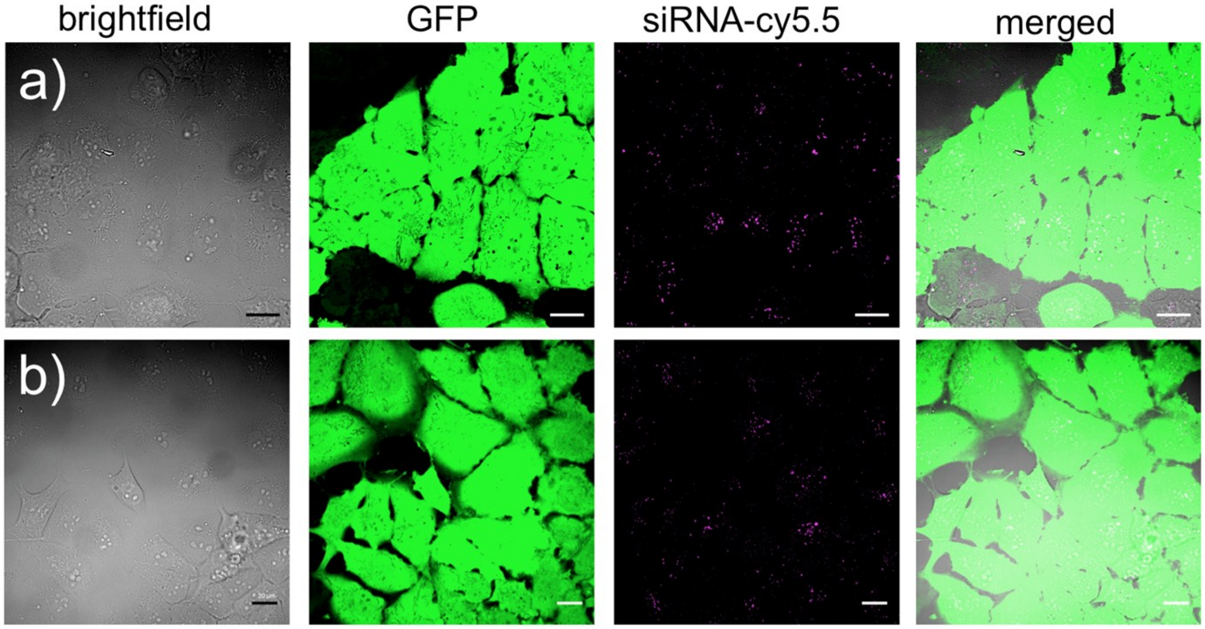

Figure S6. Confocal images of GFP expressing cells treated with NP-TCO-siRNA a) before and b) after addition of *4-isopropylbenzonitrile* which is the precursor of tetrazine **2**. The results demonstrate that the GFP signal was not lost upon treatment with *4-isopropylbenzonitrile* which was used as a control for tetrazine.

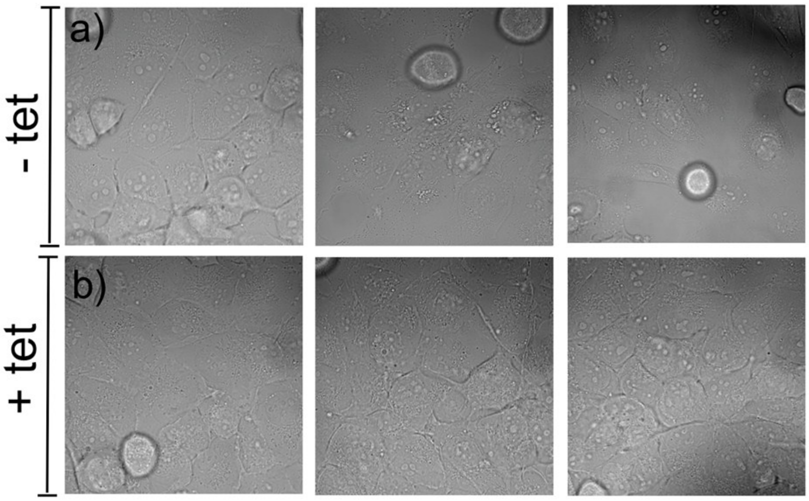

Figure S7. Bright-field images of cells a) without and b) with the addition of tetrazine **2**. Addition of the tetrazine does not induce any significant change in cellular morphology.

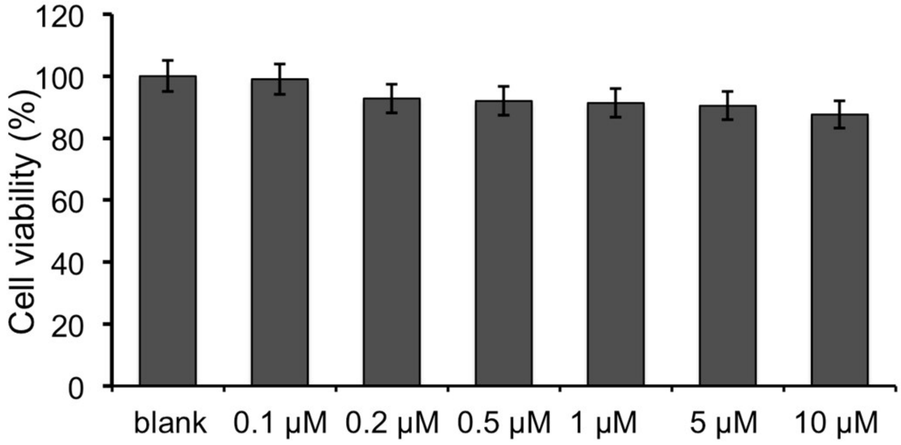

Figure S8. Cell viability data showing that there is no significant change in cytotoxicity with increasing concentrations (100 nM to 10 μM) of tetrazine.

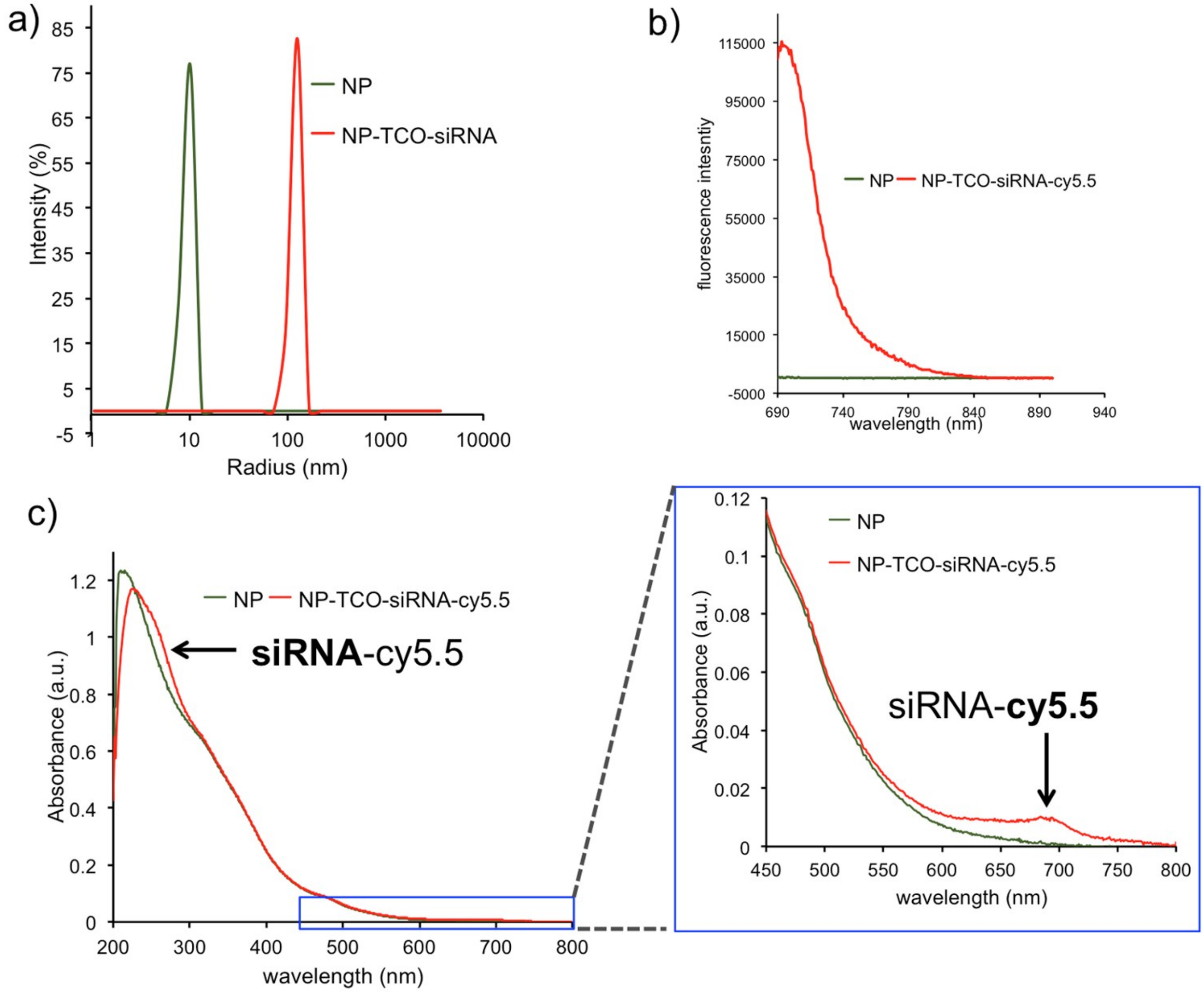

Figure S9. NP-TCO-siRNA characterization. a) DLS studies showing the increase in the hydrodynamic radius of nanoparticles after siRNA conjugation. b) Fluorescence spectra showing that NPs fluoresce around 700 nm after siRNA-cy5.5 conjugation. c) UV-Vis spectra showing that two new peaks appear after conjugation of siRNA-cy5.5 to NPs. The peak at ~260 nm was attributed to siRNA, while the peak ~690 nm in the zoomed inset to the cy5.5 label.

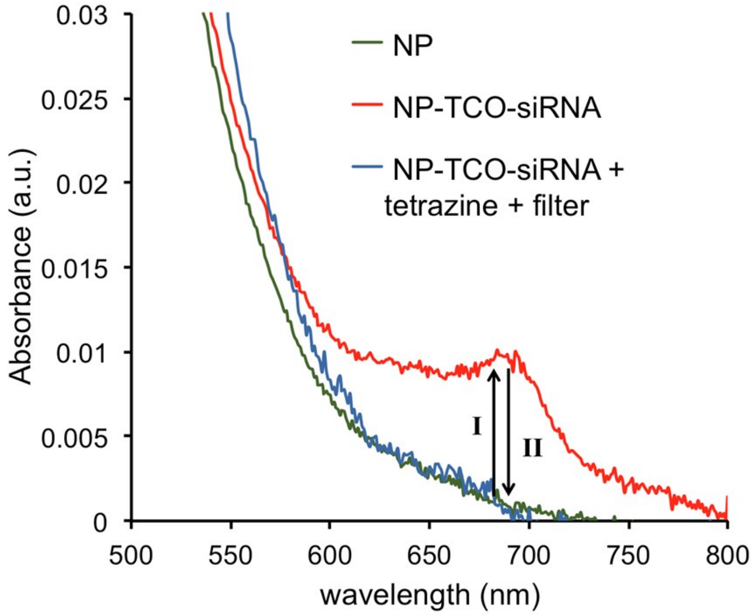

Figure S10. Conjugation (**I**) and release (**II**) of siRNA with NP. UV-Vis spectra showing that the ~690 nm cy5.5 absorbance peak appears after conjugation (**I**) of siRNA-cy5.5 to NP and disappears (**II**) upon treatment with tetrazine and filtration.
